# The Characteristics, Sources, and Health Risks of Volatile Organic Compounds in an Industrial Area of Nanjing

**DOI:** 10.3390/toxics12120868

**Published:** 2024-11-29

**Authors:** Tao Tan, Xinyuan Xu, Haixin Gu, Li Cao, Ting Liu, Yunjiang Zhang, Junfeng Wang, Mindong Chen, Haiwei Li, Xinlei Ge

**Affiliations:** 1 Management Office of Nanjing Jiangbei New Materials Science and Technology Park, Nanjing 210044, China; 2 Joint International Research Laboratory of Climate and Environment Change, Jiangsu Key Laboratory of Atmospheric Environment Monitoring and Pollution Control, Collaborative Innovation Center of Atmospheric Environment and Equipment Technology, School of Environmental Science and Engineering, Nanjing University of Information Science and Technology, Nanjing 210044, China

**Keywords:** volatile organic compounds, industrial area, emission profiles, ozone formation potential, source apportionment, health risk assessment

## Abstract

This study investigates the chemical complexity and toxicity of volatile organic compounds (VOCs) emitted from national petrochemical industrial parks and their effects on air quality in an industrial area of Nanjing, China. Field measurements were conducted from 1 December 2022, to 17 April 2023, focusing on VOC concentrations and speciations, diurnal variations, ozone formation potential (OFP), source identification, and associated health risks. The results revealed an average total VOC (TVOC) concentration of 15.9 ± 12.9 ppb and an average OFP of 90.1 ± 109.5 μg m^−3^. Alkanes constituted the largest fraction of VOCs, accounting for 44.1%, while alkenes emerged as the primary contributors to OFP, comprising 52.8%. TVOC concentrations peaked before dawn, a pattern attributed to early morning industrial activities and nighttime heavy vehicle operations. During periods classified as clean, when ozone levels were below 160 μg m^−3^, both TVOC (15.9 ± 12.9 ppb) and OFP (90.4 ± 110.0 μg m^−3^) concentrations were higher than those during polluted hours. The analysis identified the key sources of VOC emissions, including automobile exhaust, oil and gas evaporation, and industrial discharges, with additional potential pollution sources identified in adjacent regions. Health risk assessments indicated that acrolein exceeded the non-carcinogenic risk threshold at specific times. Moreover, trichloromethane, 1,3-butadiene, 1,2-dichloroethane, and benzene were found to surpass the acceptable lifetime carcinogenic risk level (1 × 10^−6^) during certain periods. These findings highlight the urgent need for enhanced monitoring and regulatory measures aimed at mitigating VOC emissions and protecting public health in industrial areas. In the context of complex air pollution in urban industrial areas, policymakers should focus on controlling industrial and vehicle emissions, which can not only reduce secondary pollution, but also inhibit the harm of toxic substances on human health.

## 1. Introduction

Volatile organic compounds (VOCs) are organic compounds having a minimum vapor pressure of 0.13 kPa at the standard temperature and pressure (293 K, 101 kPa) [1]. The substantial emission of VOCs in industrial settings underscores the significant challenges faced in enhancing urban air quality [2]. Among various pollutants, VOCs serve as critical precursors to secondary pollutants, such as ozone (O_3_) and secondary organic aerosols, thus playing a vital role in atmospheric chemical processes [3,4]. The intensive nature of industrial activities contributes to a range of environmental issues. In addition to their role as precursors to secondary pollutants, many VOCs are recognized for their harmful effects and are classified as potential carcinogens in humans [5,6]. While biogenic sources dominate global VOC emissions, high concentrations of VOCs and the associated VOC-limited O_3_ formation regime have been identified as pivotal for the synergistic control of both O_3_ and haze pollution [7,8,9,10]. Despite significant reductions in the emission of primary pollutants, such as sulfur dioxide (SO_2_), nitrogen oxides (NO_x_), and particulate matter, VOC pollution in industrial urban areas exhibits a growing trend. This persistence can be attributed to the presence of concentrated and complex emission sources, as well as the intricate speciation and chemical transformations of VOCs in these regions [11,12].

Air pollution from ozone and fine particulate matter has worsened in industrial urban areas due to complex atmospheric processes [13]. In China, increasing emissions in suburban areas, driven by the relocation of non-essential industries from downtown in urban areas, have intensified VOC pollution [14,15]. Industrial and vehicular sources have emerged as the predominant contributors to VOC emissions in these suburban areas, accounting for over 28% of the total volatile organic compound (TVOC) mixing ratio observed in urban–industrial complex metropolises [16,17]. In addition, the petrochemical industry has historically been a dominant source of VOC emissions in industrial urban settings [13]. Mitigating VOC emissions from petrochemical processes presents a promising strategy for alleviating ozone pollution [18]. Research shows that the petrochemical sector accounts for about 2630 kilotons of emissions annually, with 13.2% of total industrial VOC emissions [19]. Reducing anthropogenic VOC emissions from both industrial and vehicular sources remains a critical priority in air pollution control strategies for rapidly developing cities [15]. Although existing studies have identified the key sources and temporal patterns of VOC emissions, there is a notable gap in comprehensive assessments of their health impacts and secondary formation processes. Many investigations have focused on specific industrial sectors or geographic regions, resulting in a lack of understanding of VOC pollution across diverse urban landscapes [20,21]. Additionally, the health risks associated with VOC exposure for residents in industrial urban settings are rarely addressed, partly due to challenges in accurately estimating exposure levels and durations. Unlike risk assessments for workers, where modeling parameters such as daily exposure time (e.g., 8 h per day) can be precisely quantified, assessments for surrounding residents are considerably more complex [22]. Furthermore, existing studies on health risk assessments of VOCs predominantly focus on odor pollution associated with municipal solid waste disposal facilities [23].

Nanjing, the capital of Jiangsu Province, stands as a prominent industrialized megacity within the Yangtze River Delta (YRD) region. The Jiangbei New District (118.78 E, 32.28 N), recognized as one of the largest industrial bases in Nanjing, is home to the province’s most extensive industrial parks. There are more than 130 production and operation enterprises in the parks, including more than 20 top 500 enterprises in the world, top 50 enterprises in the global chemical industry, and leading enterprises in the market segments, such as Sinopec, BASF in Germany and Celanese in the United States, covering chemical, petrochemical, and iron and steel manufacturing industries. In light of China’s heightened emphasis on pollution prevention and control, it is imperative to deepen our understanding of VOC emissions from this area. Research focused on VOCs in industrialized urban environments will not only advance our knowledge of their sources and exposure pathways but also clarify their associated health impacts.

The aims of this work are to comprehensively investigate and analyze the concentration level, chemical composition, secondary pollution feedback, source profile, and related health risks of ambient VOCs in the environment of the national petrochemical industrial park. Utilizing the Positive Matrix Factorization (PMF) model for source identification, alongside the Potential Source Contribution Factor (PSCF) model, the source-specific health risks associated with VOCs were quantified and evaluated by estimating the Hazard Index (HI) and Lifetime Cancer Risk (LCR). These findings are intended to inform the development of effective strategies for controlling VOC emissions from industrial and vehicular sources, as well as to facilitate the elimination of the predominant harmful or toxic components in urban industrial environments. This work contributes valuable insights into the management of VOC pollution and underscores the necessity for targeted interventions to mitigate associated health risks in affected communities.

## 2. Materials and Methods

### 2.1. Sampling Location

**Figure 1:**
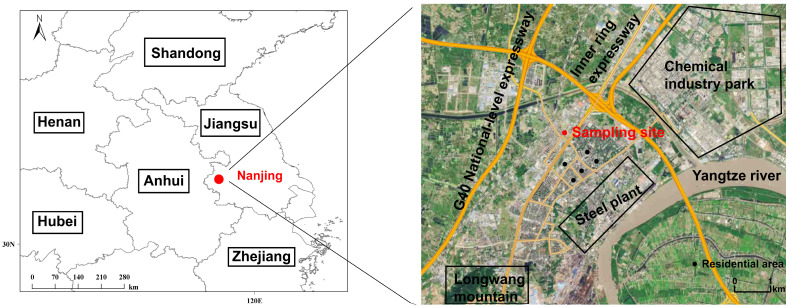
The location of the sampling site is indicated by a red dot, accompanied by a delineation of the various functional areas in the surrounding environment, including industrial, traffic, and residential areas.

As shown in Figure 1, continuous measurements were conducted at the Yongzhuang (YZ) monitoring site (118.75 E, 32.24 N) from 1 December 2022, to 17 April 2023, to investigate air pollution processes of VOCs in the national chemical industrial parks of Jiangbei New District, Nanjing. The monitoring site is located 5 km southwest of petrochemical industrial areas, 3 km southeast of traffic-rush expressways, and 4.5 km northwest of a steel plant. Residential areas are situated 1.5 km south of the sampling point, indicating that local residents may be significantly impacted by extensive emissions from both industrial and vehicular sources. This site exemplifies an industrial urban environment characterized by its proximity to factories, high traffic flows, and residential neighborhoods. Such conditions make it an ideal location for studying the interactions between industrial activities and air quality. In addition, this study categorized the measurement period into O_3_-clean days and O_3_-polluted days to facilitate a comprehensive analysis. This classification was based on the maximum daily 1 h average O_3_ concentration, with days exceeding 160 μg m^−3^, in accordance with the Class I level in China’s national ambient air quality standard, being designated as O_3_-polluted days [24].

### 2.2. Chemical Analysis

Ambient VOCs were real-time monitored using an online gas chromatograph (GC955-615/815, Synspec, The Netherlands) equipped with both a photoionization detector (PID) and a flame ionization detector (FID). The monitoring system provides data with a temporal resolution of one hour, enabling a detailed analysis of the concentration patterns and speciations of VOCs. It enables the identification of both low-boiling toxic hydrocarbons (C_2_–C_5_) and high-boiling toxic hydrocarbons (C_6_–C_12_). In total, 115 compounds were quantified, including 28 alkanes, 12 alkenes, 1 alkyne, 17 aromatic hydrocarbons, 35 halogenated hydrocarbons, 21 oxygenated volatile organic compounds (OVOCs), and 1 other substance, as listed in Table S1. It is important to note that the GC instrument is unable to accurately detect formaldehyde, the simplest C_1_ aldehyde, despite its recognition as ubiquitous human carcinogen [25].

The combination of both detectors allows for the effective monitoring of complex VOC mixtures. The PID is particularly effective for continuous monitoring of benzene, toluene, ethylbenzene, and xylenes (BTEX), as it offers high resolution and can detect concentrations as low as 150 ppt. In contrast, the FID operates effectively in the low ppb to ppm range, which has enhanced its popularity for VOC analysis. During chromatographic analysis, individual VOCs are identified based on their retention times and quantified by integrating the corresponding peaks. To ensure accuracy, the monitoring system was calibrated weekly using standard gases from the Photochemical Assessment Monitoring Stations (PAMS), in accordance with the United States Environmental Protection Agency (USEPA) TO15 standard method [13]. The detection limit for benzene is 0.1 nmol/mol, and the reproducibility was maintained below 3% for 1 ppb benzene. In addition, a multi-point calibration was conducted quarterly, with standard gases diluted to concentrations of 0, 1, 2, 5, 10, 20, and 40 ppb for a 7-point calibration. The correlation coefficient (R^2^) of the calibration curve had to exceed 0.99, and each calibration was validated by passing the standard gas at least five times until equilibrium was achieved. Rigorous quality control measures are implemented to validate the results and address any potential anomalies or losses due to instrument maintenance or malfunctions. Additional trace gases such as NO_x_ (NO + NO_2_), O_3_, and CO, as well as key meteorological parameters including temperature, relative humidity (RH), wind speed, and wind direction, were measured at one-minute intervals at the air quality monitoring station.

### 2.3. Data Analysis

#### 2.3.1. Ozone Formation Potential (OFP)

Maximum incremental reactivity (MIR) is a key metric for evaluating the *OFP* of VOCs [26]. This approach specifically assesses the dynamics of ozone production in relation to variations in VOC concentrations [27]. The formula for calculating *OFP* is presented in the following Equation (1):

(1) $$OFP={\left[VOC\right]}_{i}\times MIR_{i}$$

where [*VOC*]*_i_* represents the mass concentration of VOC species *i* in the atmosphere (μg m^−3^), and the MIR coefficient (*MIR_i_*) for each VOC is determined by the molecular mass ratio of O_3_ to *VOC_i_* (g_O3_/g*_VOCs_*), which can be sourced from existing literature [28].

#### 2.3.2. Ozone Sensitivity Analysis

O_3_ is generated through atmospheric photochemical reactions involving NO_x_ and VOCs in specific ratios [29]. Understanding the role of these precursors in O_3_ formation is critical, as the process can be characterized by either VOC-limited or NO_x_-limited formation [30]. Identifying whether a region is primarily constrained by VOCs or NO_x_ is essential for effective strategies to mitigate regional ozone pollution. The ratio of VOCs to NO_x_ serves as a valuable tool for elucidating the mechanisms driving ozone formation in a specific area.

#### 2.3.3. Source Apportionment

Positive Matrix Factorization (PMF) analysis is a widely employed tool for source apportionment in air quality studies [31]. This method is grounded in the principle of mass conservation, enabling it to function without requiring prior knowledge of specific parameters for each source or temporal trends. PMF operates under the constraint that the characteristic spectrum and contribution values of each source must be non-negative [32]. Typically, PMF is applied to long-term observational datasets with low temporal resolution, allowing for the identification and quantification of the primary sources of VOCs in a region. Here, the PMF 5.0 developed by the USEPA was utilized to systematically identify the sources of VOCs. The PMF model requires the provision of concentration data and uncertainty data, with the uncertainty calculated as follows by Equations (2) and (3) [33]:

(2) $$Unc=\sqrt{{\left(EF\times c\right)}^{2}+{\left(0.5\times MDL\right)}^{2}}\;\left(c>MDL\right)$$

(3) $$Unc=(\frac{5}{6})\times MDL\;(c\leq MDL)$$

where *Unc* represents uncertainty, *EF* denotes the error fraction (%), *c* refers to the concentration of the species, and *MDL* stands for the method detection limit. A group of 27 major species of VOCs was input into the model analysis, which are representative traces for different sources with relatively high abundance and S/N value. The measured data below MDLs was substituted with MDL/2 and missing data was assigned the median concentration.

In addition, Potential Source Contribution Factor (PSCF) analysis was employed to identify potential pollution sources based on principles of conditional probability [34]. By integrating results from backward trajectory simulations, the PSCF generates a high-resolution grid over the study area, tracking pollution trajectories when concentrations exceed a specified threshold. This method facilitates a detailed examination of pollution sources and their spatial distribution, with higher PSCF values indicating a greater likelihood of contributing to VOC pollution [35,36]. In this study, we utilized hourly pollutant concentration data collected from 1 December 2022, to 17 April 2023, in conjunction with the Global Aerosol Data Set (GADS) meteorological data. The Hybrid Single Particle Lagrangian Integrated Trajectory (HYSPLIT) model was employed for backward trajectory simulations of atmospheric pollutants in Jiangbei New District, set at a model run height of 500 m and a simulation duration of 36 h, and conducted hourly from 00:00 to 23:00 (UTC) each day. Utilizing TrajStat software (MeteoInfo 3.9), we clustered the air mass trajectories from this period into four categories using the Euclidean distance method, which enabled a comprehensive analysis of the transport routes and distribution patterns of environmental air pollutants [37,38].

#### 2.3.4. Risk Characterization

The hazard index (*HI*) and the lifetime cancer risk (*LCR*) corresponding to the residents’ non-carcinogenic and carcinogenic risks to human health were estimated to evaluate the health risks of VOC species as follows [39,40,41,42]:

(4) $$LCR={\left[VOC\right]}_{mi}\times \frac{ET\times EF\times ED}{AT}\times IUR_{i}$$

(5) $$LCR=\sum LCR_{i}$$

(6) $$NCR_{i}={\left[VOC\right]}_{mi}\times \frac{\frac{ET\times EF\times ED}{AT*1000}}{RfC_{i}}$$

(7) $$HI=\sum NCR_{i}$$

where the daily exposure time (*ET*), exposure frequency (*EF*), and exposure duration (*ED*) for residents were approximately 3.1 h day^−1^, 365 days year^−1^, and 76.4 years, respectively, which were obtained from the Exposure Factors Handbook of Chinese Population (Adults) [43]. The *AT* is the average exposure time and was set at (365 × 76.4 × 24) hours. The values of inhalation unit cancer risk (*IUR_i_*, m^3^ μg^−1^) and reference concentrations (*RfC_i_*, μg m^−3^) of VOC species were referenced from the Integrated Risk Information System (IRIS), USEPA [44]. The values of *RfCi* and *IURi* used for the health risk assessment are introduced in Tables S2 and S3.

## 3. Results and Discussion

### 3.1. Concentration Level and Composition Characteristics of VOCs

**Figure 2:**
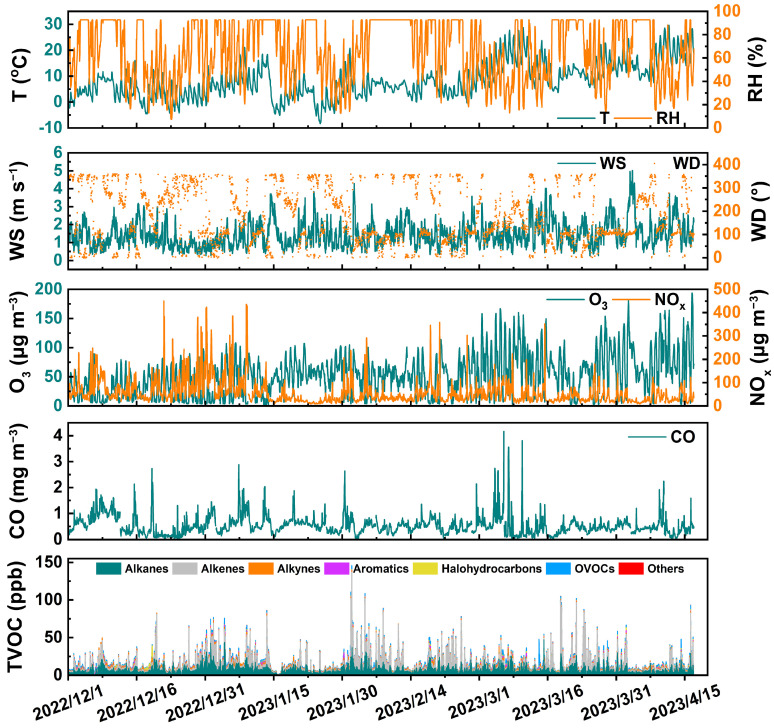
Time series of variations in concentrations of atmospheric gaseous pollutants and meteorological parameters.

On average, the TVOC concentrations were 15.9 ± 12.9 ppb during this summer campaign, as shown in Figure 2. The average temperature was 8.5 ± 6.7 °C and was associated with a wind speed of 1.5 ± 0.7 m s^−1^; the average humidity reached 66.3 ± 24.8%; the north wind prevailed in the winter; and the southeast wind and east wind prevailed in the spring; thereby, the meteorological conditions were characterized by low temperatures and low wind speeds, which is in favor of the local accumulation of air pollutants. Figure S1 illustrates that alkanes accounted for approximately 44.1% of the total VOC contribution, with a concentration of 7.0 ± 4.5 ppb. Alkenes followed, contributing 31.3% at 5 ± 8.7 ppb, while oxygenated volatile organic compounds (OVOCs) comprised 9.1% (1.4 ± 1.4 ppb) and alkynes accounted for 8.2% (1.3 ± 0.8 ppb). Alkanes emerged as the predominant VOCs, consistent with findings in other economically developed cities in China, such as Beijing [45], Shanghai [46], and Wuhan [47,48]. The TVOC concentrations observed at industrial areas in Nanjing were comparable to levels found in certain southeastern regions of China near industrial parks [49]. However, the TVOC concentrations in Kaohsiung, which hosts Taiwan’s largest heavy industrial area, and in Dongguan, an industrially developed city, were notably higher than those recorded in this study [50,51]. The variations in VOC emissions across different regions can be attributed to several factors, including geographical and climatic conditions, analytical methodologies, and the specific industrial composition of each area. Figure S2 presents the top ten VOCs by concentration, with ethylene recorded at 3.7 ± 7 ppb, and closely followed by ethane at 3.1 ± 1.4 ppb. These elevated levels are likely associated with emissions from the petrochemical industries. The petrochemical sector, a downstream component of petroleum refining, utilizes oil and gas as raw materials to produce essential products such as olefins and aromatics, which are critical for the broad chemical industry [52]. VOC emissions from this sector primarily stem from combustion exhaust and process tail gases, with key pollutants including benzene, toluene, xylene, and ethylene [44,53]. Additionally, ethylene is present in vehicle exhaust, highlighting the diverse sources of VOCs in industrial urban areas [14].

In addition, the concentrations of O_3_ averaged 51.7 ± 35.1 μg m^−3^, while the average concentration of NO_x_ was 56.2 ± 50.9 μg m^−3^. The variations in the concentrations of VOCs and NO_x_ exhibited a similar trend. When the concentration of O_3_ increases, it is often accompanied by a decrease in the concentrations of VOCs and NO_x_, occurring during periods of higher temperatures and lower humidity. However, due to the existence of complex chemical reaction processes between pollutants and radicals, the net production and loss of tropospheric O_3_ is not proportional to the mitigation of precursor VOCs and NO_x_, and is affected by the reaction rates of hydroperoxyl radicals (HO_2_) and peroxy radicals (RO_2_) [21]. Throughout the entire observation period, the hourly average concentration of O_3_ achieved a compliance rate of approximately 99.6%, based on its standardized first-level concentration limit of 160 µg m^−3^. The hourly average concentration of CO was 0.5 ± 0.4 mg m^−3^, which remained well below the first-level concentration limit of 10 mg m^−3^.

**Figure 3:**
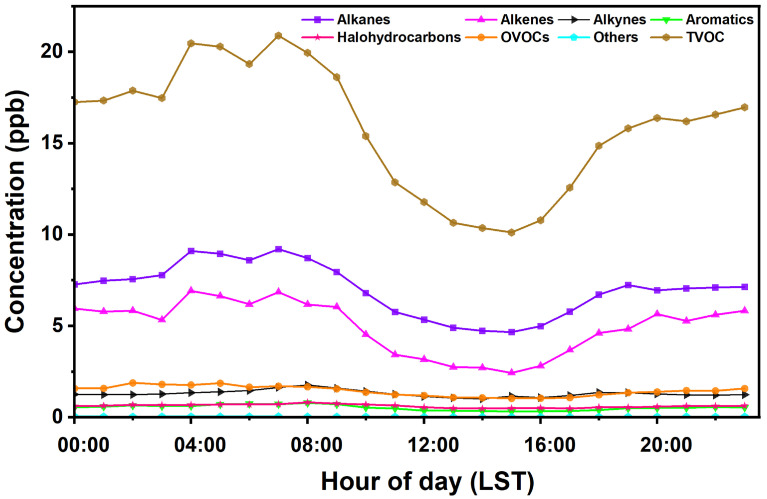
Diurnal variations in concentration of different categories of VOC groups.

As shown in Figure 3, the diurnal variations of VOCs exhibited a distinct pattern, characterized by an initial peak occurring just before dawn. Both alkanes and alkenes demonstrated similar trends, which may be attributed to the presence of numerous chemical enterprises near the observation point that typically operate during the early morning hours. Additionally, nighttime traffic restrictions in urban areas often necessitate the operation of construction vehicles at night, leading to significant emissions of vehicle exhaust during the early morning. Following this initial peak, influenced by motor vehicle emissions and the cooler temperatures of early morning, VOC concentrations reached their daily maximum between 7:00 and 8:00 a.m. As sunlight intensified throughout the day, the VOC levels gradually declined. During midday, reduced traffic and active photochemical processes, coupled with rising temperatures and an increase in the height of the atmospheric boundary layer, enhanced near-surface wind speeds. These conditions facilitated the dispersion of VOCs, resulting in lower concentrations observed from 2:00 to 3:00 p.m. In the evening, an uptick in traffic emissions, combined with a decrease in the atmospheric boundary layer height, impeded the dissipation of VOCs. This led to a gradual accumulation of these compounds, resulting in sustained higher concentration levels. Overall, the diurnal cycle of VOCs reflects the interplay between emissions from industrial activities and vehicular traffic, as well as the significant influence of meteorological factors on atmospheric chemistry.

### 3.2. Chemical Reactivity Evaluation

**Figure 4:**
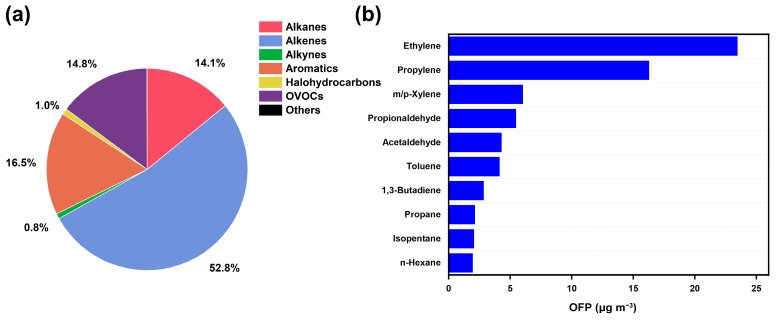
Variations in contribution of major categories of VOCs to OFP (**a**) and comparisons in top ten VOC species contributing to OFP (**b**).

As shown in Figure 4, the concentration of OFP attributed to the TVOCs was estimated at 90.2 ± 109.5 μg m^−3^. The OFP was predominantly composed of olefins, which accounted for 52.8% of the total, followed by aromatics at 16.5%. OVOCs and alkanes contributed 14.8% and 14.1%, respectively. The top ten species influencing the OFP were identified based on their maximum incremental reactivity coefficients. Identified as a common volatile compound produced by petrochemical industries, ethylene exhibited the highest OFP value at 23.5 ± 44.8 μg m^−3^, followed by propylene at 16.3 ± 52.3 μg m^−3^. Notably, ethylene alone accounted for 26.0% of the total OFP and 31.0% of the overall TVOC levels, underscoring the importance of controlling olefins to mitigate ozone pollution. As companion emitters from industrial solvents used in chemical production [54], acetaldehyde and propionaldehyde made substantial contributions to the OFP despite their lower concentration levels of 0.4 ± 0.3 ppb and 0.3 ± 0.4 ppb, respectively. In contrast, acetone, which exhibited the highest concentration level among the OVOCs at 0.6 ± 0.8 ppb, did not contribute comparably to the OFP as acetaldehyde and propionaldehyde did.

**Figure 5:**
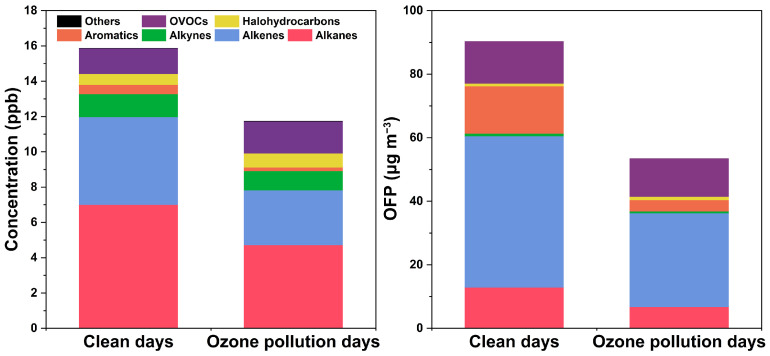
Comparison of VOC concentration and ozone formation potential between ozone pollution days and clean days.

Figure 5 shows the differences in the TVOC concentrations and OFP between clean days, characterized by lower ozone pollution, and periods of higher pollution. On clean days, the average TVOC concentration was 15.9 ± 12.9 ppb, with alkanes and alkenes dominating at 44.0% and 31.3%, respectively. OVOCs and acetylenes together accounted for 17.2%. The average total OFP during these periods was 90.4 ± 110.0 μg m^−3^, with alkenes contributing the largest share at 52.9%, while alkanes, aromatics, and OVOCs contributed 14.1%, 16.5%, and 14.7%, respectively. During periods of elevated ozone pollution, the average TVOC concentration decreased to 111.0 ± 4.3 ppb, representing a reduction of 4.8 ppb compared to clean days. Alkanes remained the predominant species at 43.1%, followed by olefins at 19.4% and OVOCs at 17.3%, with other compounds comprising 20.2%. The average total OFP during pollution hours was 47.3 ± 32.1 μg m^−3^, with alkenes and OVOCs contributing 44.4% and 27.0%, respectively, while alkanes contributed less at 14.8%. These findings indicate that both TVOC concentrations and total OFP values are generally higher during clean hours compared to pollution hours. Throughout both periods, alkanes consistently represented the largest proportion of VOCs, while alkenes contributed significantly to the OFP during clean hours. Table S4 provides a comparison of pollutant concentrations and meteorological conditions on clean days versus ozone pollution days. Notably, the occurrence of ozone pollution in the industrial areas showed no alleviated trends under relatively high wind speed conditions, despite a decrease in TVOC emissions. While higher temperatures typically promote local ozone formation [55], the regional transport of pollutants significantly exacerbates ozone pollution [56]. This transport can counteract efforts to reduce primary pollutant emissions, complicating the overall mitigation of air quality issues.

It is generally believed that the critical value of ozone-sensitive area attribute diagnosis is 8. When the ratio of VOC/NOx (VOCs use carbon content, NOx uses volume fraction) is less than 4, ozone formation is controlled by VOCs. When it is greater than 15, ozone formation is controlled by NOx, while a ratio between 4 and 15 belongs to the transition zone [57]. A numerical simulation of ozone pollution in Los Angeles indicated that when the VOC to NO_x_ ratio approaches 8:1, the area shifts from VOC-limited to NO_x_-limited conditions [58]. As shown in Figure S3, the average VOC/NO_x_ ratio (ppbC/ppbv) remained consistently below 8, with 90% of the data points falling under this threshold. This suggests that ozone formation is sensitive to VOC levels and currently operates within a VOC-controlled regime.

### 3.3. Potential Source Identification

#### 3.3.1. PMF Model Analysis

**Figure 6:**
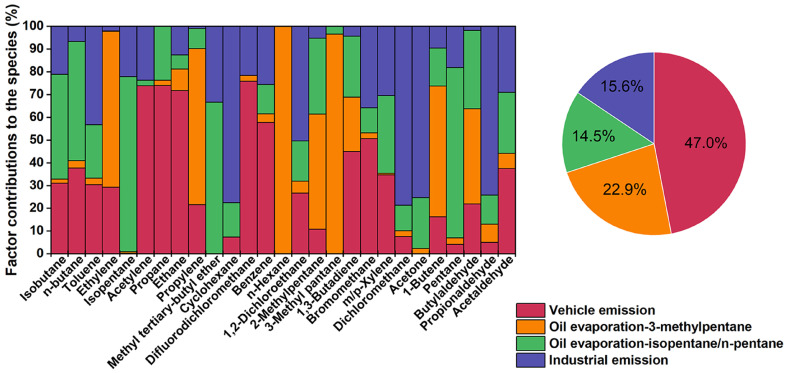
Relative contributions of PMF-resolved sources to TVOCs.

Figure 6 presents the classification of VOC sources. Factor 1 is notably characterized by high concentrations of ethane, benzene, propane, and acetylene. Acetylene, often used as a tracer for vehicle exhaust, alongside C_3_–C_5_ olefins and alkanes, indicates significant contributions from traffic emissions due to the incomplete combustion of gasoline and diesel. Thus, this factor is identified as vehicle exhaust [59]. Factor 2 is dominated by 3-methylpentane, which constitutes 96.5% of this factor, along with n-hexane, contributing the remainder of the 100%. The prevalence of 3-methylpentane is closely linked to gasoline evaporation, leading to its classification as oil and gas evaporation–3-methylpentan [60]. Factor 3 features high levels of isopentane, n-pentane, and methyl tert-butyl ether (MTBE), with contributions of 77%, 74.8%, and 66.6%, respectively. MTBE, a common gasoline additive, serves as a marker for gasoline-related emissions, thus categorizing this factor as oil and gas evaporation–isopentane/n-pentane [61,62]. Therefore, Factor 3 is identified as oil and gas evaporation–isopentane/n-pentane. Finally, Factor 4 is primarily composed of dichloromethane, which accounts for 78.6%. Compounds like dichloromethane and cyclohexane are associated with industrial solvents, leading to the classification of this factor as industrial emissions, particularly given the proximity of several factories to the sampling site [63,64]. According to the source apportionment results, vehicle exhaust had the highest contribution to ambient VOCs (47%), followed by oil and gas evaporation–isopentane/n-pentane (22.9%), industrial emission (15.6%), and oil and gas evaporation–3-methylpentan (14.5%). Therefore, in order to improve the VOCs pollution situation in the region, it is necessary to focus on controlling road mobile sources and petrochemical enterprises.

#### 3.3.2. Potential Source Contribution Analysis

**Figure 7:**
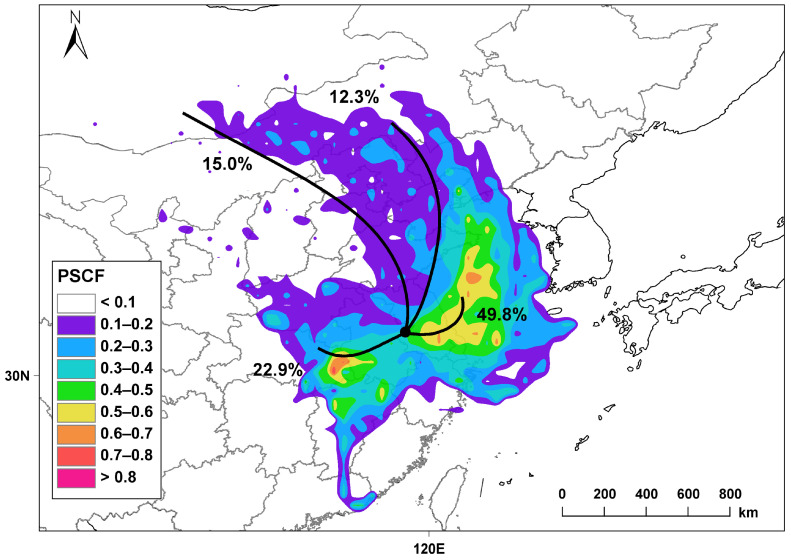
The 24 h backward trajectories and potential source contribution functions (PSCFs) for TVOCs.

Air mass trajectory clustering identified four primary pathways based on the PSCF analysis (Figure 7). The first pathway originates from the central Yellow Sea, traversing Taizhou and Zhenjiang in Jiangsu before converging in Nanjing. This medium-distance air mass accounts for the largest proportion at 49.8%, establishing it as the primary route for pollution transport to the observation point due to its relatively short transmission distance. The second pathway begins in Xiaogan, Hubei Province, and moves through Anhui Province before reaching Nanjing, representing a medium-long distance air mass with a clustering proportion of 22.9%. In addition, two long-distance air masses originate from Inner Mongolia, with clustering proportions of 15% and 12.3%, respectively. The areas with PSCF values > 0.5 were mainly distributed in the eastern and southwestern parts of Nanjing, including some cities in Jiangsu and Hubei, so the VOCs in Nanjing were greatly affected by these potential sources. Given these findings, it is essential to prioritize VOC prevention and reduction measures in these identified regions, particularly along the primary and secondary air mass pathways. Implementing targeted strategies in these areas can significantly mitigate the impact of pollution transport to Nanjing and enhance air quality management efforts.

### 3.4. Health Risk Assessment

**Figure 8:**
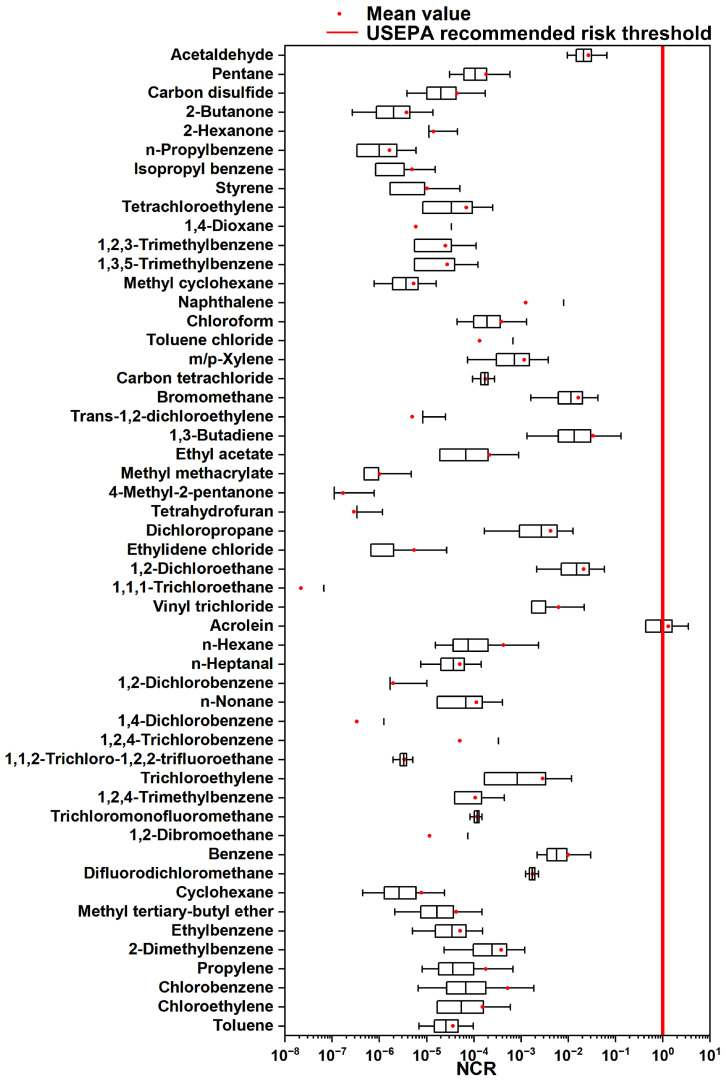
Assessment of non-carcinogenic risk (NCR) index via inhalation exposure of nearby residents to VOCs.

**Figure 9:**
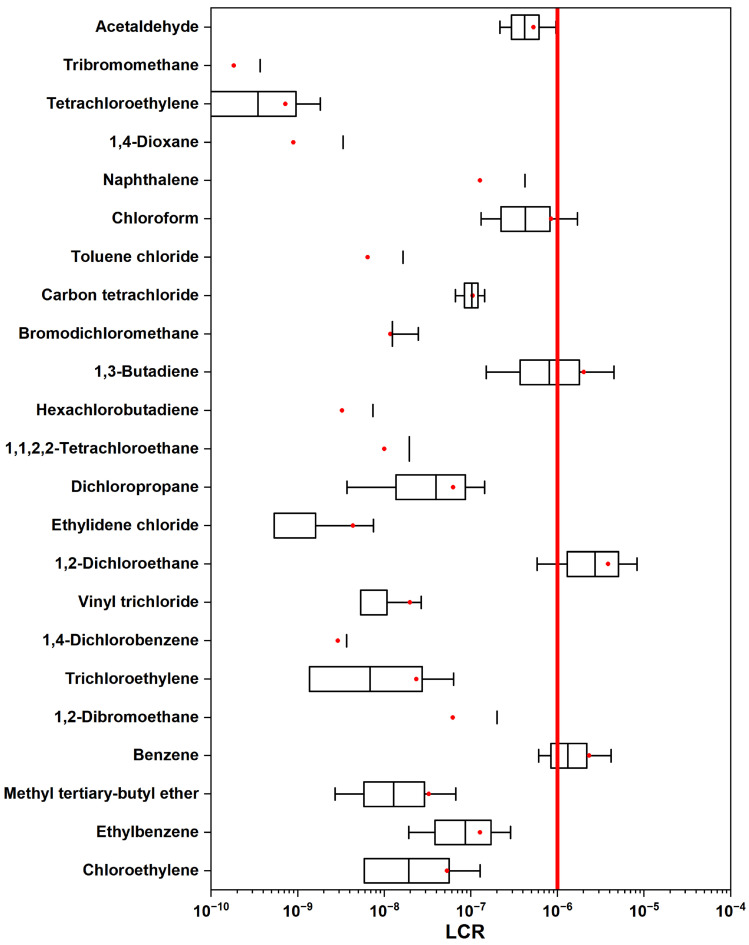
Assessment of lifetime carcinogenic risk (LCR) index via inhalation exposure of nearby residents to VOCs.

As shown in Figure 8, the HI value of only acrolein (1.3 ± 2.2) exceeded the non-carcinogenic health risk threshold (NCR > 1) recommended by the USEPA, while all other compounds were still below the recommended level, but some were also worthy of attention, such as bromomethane (1.6 ± 2.6 × 10^−2^), 1,2-dichloroethane (2.1 ± 2.2 × 10^−2^), 1,3-butadiene (3.4 ± 7.2 × 10^−2^), and acetaldehyde (2.7 ± 1.9 × 10^−2^). Acrolein, a significant chemical precursor or biocide widely used in the petrochemical industries, can be a strong irritant for the skin, eyes, and nasal passages, and long-term exposure can even cause a risk of cancer [65]. Studies have found that acetaldehyde is a non-carcinogenic risk substance in the VOC emissions of the recycled rubber industry [66] and 1,3-butadiene has a non-carcinogenic risk in petrochemical industrial areas [22]. Similarly, the cancer risk assessment results are presented in Figure 9. During specific time frames, three critical compounds, including 1,3-butadiene (2.0 ± 4.3 × 10^−6^), 1,2-dichloroethane (3.9 ± 4.1 × 10^−6^), and benzene (2.3 ± 4.2 × 10^−6^), exceeded the USEPA’s recommended cancer risk threshold (LCR > 1 × 10^−6^) [67,68]. It should be noted that their corresponding concentration levels were relatively low, at 0.1 ± 0.2, 0.1 ± 0.1, and 0.3 ± 0.5 ppb, respectively. This underscores their significant contributions to the cancer risk in typical industrial areas. The health risk assessment, which evaluated 151 VOCs over nearly five months during the cold season of a typical year, indicates that both extensive emissions and air stagnation could lead to more severe health risks for the surrounding environment and the residents. The findings from both the non-cancer and cancer risk assessments underscore the urgent need for measures to control pollutant emissions.

## 4. Conclusions

Significant emissions were identified as being closely associated with local chemical activities. The diurnal variation analysis indicated that the first concentration peak occurred just before dawn and was driven by nearby industrial operations and nighttime construction activities. Notably, the nighttime concentrations surpassed those observed during the day, with the lowest levels recorded in the afternoon. The average OFP was calculated at 90.2 ± 109.5 μg m^−3^, which was primarily attributed to ethylene, followed by propylene. Interestingly, the TVOC and total OFP values were higher during clean hours (15.9 ± 12.9 ppb and 90.4 ± 110 μg m^−3^, respectively) compared to pollution hours, suggesting that ozone generation occurred within the VOC control area. Source apportionment identified three primary sources of VOCs: vehicle exhaust, oil vapor evaporation, and industrial emissions, with vehicle exhaust being the most significant contributor. In terms of health risks, the HI value in this area exceeded the recommended non-carcinogenic risk value, and acrolein was the only compound that exceeded this value. For lifetime cancer risk, chloroform, 1,3-butadiene, 1,2-dichloroethane, and benzene also surpassed the threshold (1 × 10^−6^) at certain times. This study provides a comprehensive analysis of VOC emissions in an industrial area, focusing on their concentrations, sources, and associated health risks. It emphasizes the importance of monitoring and mitigation strategies for air pollution. However, the study has limitations that should be acknowledged. The observation period may not encompass seasonal variations in VOC emissions, which can fluctuate significantly due to changing industrial activities and environmental conditions. Additionally, this research was confined to a specific industrial area, potentially limiting the generalizability of the findings to other regions. While PMF effectively identifies primary sources, it may not account for all potential contributors to VOC emissions. Furthermore, the assessment was restricted to certain VOCs, possibly overlooking others that pose significant health risks. These limitations may hinder the overall understanding of VOC emission trends and underestimate the pollution burden, thereby affecting the effectiveness of mitigation strategies and public health interventions. This study offers an in-depth examination of VOC emissions within a typical urban industrial area, concentrating on their levels, origins, and related health hazards. It highlights the critical need for monitoring and strategies to reduce air pollution. The findings provide essential information for managing VOC emissions and stress the importance of implementing targeted measures to alleviate health risks in impacted communities.

## Data Availability

The data are available from the corresponding author upon reasonable request.

## Supplementary Materials

The following supporting information can be downloaded at: https://www.mdpi.com/article/10.3390/toxics12120868/s1. Supplementary Information (SI) associated with this article can be found in this section: Table S1: A list for the total of 115 VOC species measured by the GC system; Table S2: The values of reference concentrations of VOC species for non-carcinogenic risk (NCR) assessment [69]; Table S3: The values of inhalation unit cancer risk (IUR) of VOC species for lifetime carcinogenic risk (LCR) assessment [69]; Table S4: Comparisons of main meteorological parameters and concentrations of ozone, TVOCs, and OFP on clean days with those on ozone pollution days; Figure S1: Chemical composition of VOCs species; Figure S2: The top ten VOC species by concentration; Figure S3:The daily average ratio of VOCs/NO_x_.
