# Supplementary material for: The Characteristics, Sources, and Health Risks of Volatile Organic Compounds in an Industrial Area of Nanjing
Source: Toxics. 2024 Nov 29;12(12):868. doi: 10.3390/toxics12120868 (PMC11679105; doi:10.3390/toxics12120868)
Supplement: Supplementary file 1 [file toxics-12-00868-s001.zip › toxics-3319311-supplementary.pdf]

Supplementary materials

The Characteristics, Sources, and Health Risks of Volatile Organic Compounds in an Industrial Area of Nanjing

Tao Tan [1,†], Xinyuan Xu [2,†], Haixin Gu [2], Li Cao [2], Ting Liu [2], Yunjiang Zhang [2], Junfeng Wang [2], Mindong Chen [2], Haiwei Li [2,*] and Xinlei Ge [2,*]

[1] Management office of Nanjing Jiangbei New Materials Science and Technology Park,
Nanjing 210044, China; tant@ncip.cn

[2] Joint International Research Laboratory of Climate and Environment Change, Jiangsu Key Laboratory of Atmospheric Environment Monitoring and Pollution Control, Collaborative Innovation Center of
Atmospheric Environment and Equipment Technology, School of Environmental Science and Engineering, Nanjing University of Information Science and Technology, Nanjing 210044, China;
xuxiny0205@163.com (X.X.); guhaixin0515@163.com (H.G.);
20211248055@nuist.edu.cn (L.C.); 202283280008@nuist.edu.cn (T.L.);
yjzhang@nuist.edu.cn (Y.Z.); wangjunfengnuist@163.com (J.W.);
chenmd@nuist.edu.cn (M.C.)

* Correspondence: authors: haiwei.li@nuist.edu.cn (H.L.); caxinra@163.com (X.G.)

† These authors contributed equally to this work.

Table S1. A list for the total of 115 VOC species measured by the GC system.

Category of VOCs	VOC Species
Alkanes (28)	n-Butane, isopentane, cyclopentane, propane, ethane, cyclohexane, n-nonane, n-heptane, n-hexane, n-undecane, n-dodecane, 2,2,4-trimethylpentane, 2-methylpentane, 2-methylhexane, 2,3-dimethylpentane, 2,4-dimethylpentane, 3-methylpentane, n-octane, methylcyclopentane, 2,3-dimethylbutane, 2,2-dimethylbutane, 3-methylheptane, 2-methylheptane, 2,3,4-trimethylpentane, 3-methylhexane, methylcyclohexane, n-decane, n-pentane
Alkenes (12)	Isobutane, ethylene, trans-2-pentene, cis-2-pentene, 1-pentene, propylene, trans-2-butene, cis-2-butene, 1-hexene, 1,3-butadiene, isoprene, 1-butene
Alkynes (1)	Acetylene
Aromatic hydrocarbons (17)	Toluene, o-xylene, ethylbenzene, benzene, p-diethylbenzene, 1,3-diethylbenzene, 1-ethyl-3-methylbenzene, p-ethyltoluene, 1,2,4-trimethylbenzene, 1-ethyl-2-methylbenzene, m/p-xylene, naphthalene, 1,3,5-trimethylbenzene, 1,2,3-trimethylbenzene, styrene, cumene, n-propylbenzene
Halogenated hydrocarbons (35)	Vinyl chloride, chlorobenzene, difluorodichloromethane, 1,2-dibromoethane, trichlorofluoromethane, trichloroethylene, 1,1,2,2-tetrafluoro-1,2-dichloroethane, 1,1,2-trichloro-1,2,2-trifluoroethane, 1,2,4-trichlorobenzene, 1,1-dichloroethylene, 1,4-dichlorobenzene, 1,3-dichlorobenzene, 1,2-dichlorobenzene, 1,1,2-trichloroethane, 1,1,1-trichloroethane, 1,2-dichloroethane, 1,1-dichloroethane, chloroethane, dichloromethane, 1,2-dichloropropane, 1,1,2,2-Tetrachloroethane, hexachlorobutadiene, trans-1,3-dichloropropene, cis-1,2-dichloroethylene, trans-1,2-dichloroethylene, bromomethane, bromodichloromethane, dibromochloromethane, carbon tetrachloride, chlorotoluene, trichloromethane, dichloromethane, cis-1,3-dichloropropene, tetrachloroethylene, tribromomethane
OVOCs (21)	Methyl tertiary-butyl ether, acrolein, vinyl acetate, tetrahydrofuran, 4-methyl-2-pentanone, methyl methacrylate, ethyl acetate, isopropanol, 1,4-dioxane, acetone, 2-hexanone, 2-butanone, benzaldehyde, hexanal, isobutenal, n-butanal, valeraldehyde, butenal, propionaldehyde, 3-methylbenzaldehyde, acetaldehyde
Other (1)	Carbon disulfide

Table S2. The values of reference concentrations of VOC species for non-carcinogenic risk (NCR) assessment [1].

VOC species	Reference concentrations (Rfc, μg m^{-3})
Toluene	5
Chloroethylene	0.08
Chlorobenzene	0.05
Propylene	3
2-Dimethylbenzene	0.1
Ethylbenzene	1
Methyl tertiary-butyl ether	3
Cyclohexane	6
Difluorodichloromethane	0.1
Benzene	0.03
1,2-Dibromoethane	0.009
Trichloromonofluoromethane	0.7
1,2,4-Trimethylbenzene	0.06
Trichloroethylene	0.002
1,1,2-Trichloro-1,2,2-trifluoroethane	5
1,2,4-Trichlorobenzene	0.002
1,4-Dichlorobenzene	0.8
n-Nonane	0.02
1,2-Dichlorobenzene	0.2
n-Heptanal	0.4
n-Hexane	0.7
Acrolein	0.00002
Vinyl trichloride	0.0002
1,1,1-Trichloroethane	5
1,2-Dichloroethane	0.007
Ethylidene chloride	0.5
Dichloropropane	0.004
Tetrahydrofuran	2
4-Methyl-2-pentanone	3
Methyl methacrylate	0.7
Ethyl acetate	0.07
1,3-Butadiene	0.002
Trans-1,2-dichloroethylene	0.04
Bromomethane	0.005
Carbon tetrachloride	0.1
m/p-Xylene	0.1
Toluene chloride	0.001
Chloroform	0.098
Naphthalene	0.003
Methyl cyclohexane	3
1,3,5-Trimethylbenzene	0.06
1,2,3-Trimethylbenzene	0.06
1,4-Dioxane	0.03
Tetrachloroethylene	0.04
Styrene	1
Isopropyl benzene	0.4

n-Propylbenzene	1
2-Hexanone	0.03
2-Butanone	5
Carbon disulfide	0.7
Pentane	1
Acetaldehyde	0.009

[1] U.S. EPA (United States Environmental Protection Agency) Indoor air quality (IAQ): technical overview of volatile organic compounds[R]. 2017.

Table S3. The values of inhalation unit cancer risk (IUR) of VOC species for lifetime carcinogenic risk (LCR) assessment [1].

VOC species	Inhalation unit cancer risk (IUR, $m^3\ \mu g^{-1}$)
Chloroethylene	0.0000044
Ethylbenzene	0.0000025
Methyl tertiary-butyl ether	0.00000026
Benzene	0.0000078
1,2-Dibromoethane	0.0006
Trichloroethylene	0.0000041
1,4-Dichlorobenzene	0.000011
Vinyl trichloride	0.000016
1,2-Dichloroethane	0.000026
Ethylidene chloride	0.0000016
Dichloropropane	0.0000037
1,1,2,2-Tetrachloroethane	0.000058
Hexachlorobutadiene	0.000022
1,3-Butadiene	0.00003
Bromodichloromethane	0.000037
Carbon tetrachloride	0.000006
Toluene chloride	0.000049
Chloroform	0.000023
Naphthalene	0.000034
1,4-Dioxane	0.000005

Tetrachloroethylene	0.00000026
Tribromomethane	0.0000011
Acetaldehyde	0.0000022

[1] U.S. EPA (United States Environmental Protection Agency) Indoor air quality (IAQ): technical overview of volatile organic compounds[R]. 2017.

Table S4. Comparisons of main meteorological parameters and concentrations of ozone, TVOCs, and OFP on clean days with those on ozone pollution days.

**Type of ozone pollution**	**Wind speed (m s^{-1})**	**Temperature (℃)**	**Relative humidity (%)**	**O_3-1h ($\mu g \cdot m^{-3}$)**	**TVOCs (ppb)**	**OFP ($\mu g\ m^{-3}$)**
Clean days	0.6 ± 0.7	5.3 ± 6.7	22.0 ± 24.7	27.6±34.2	15.9 ± 12.9	90.3 ± 109.7
Ozone pollution days	2.0 ± 0.8	26.3 ± 1.8	37.8 ± 6.9	173.0±10.5	11.1 ± 4.3	53.5 ± 55.5

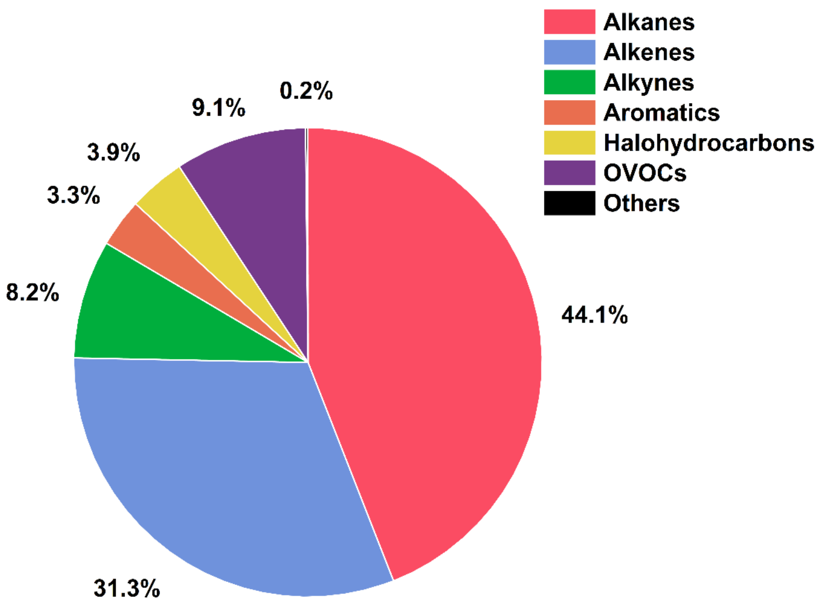

Figure. S1. Chemical composition of VOCs species.

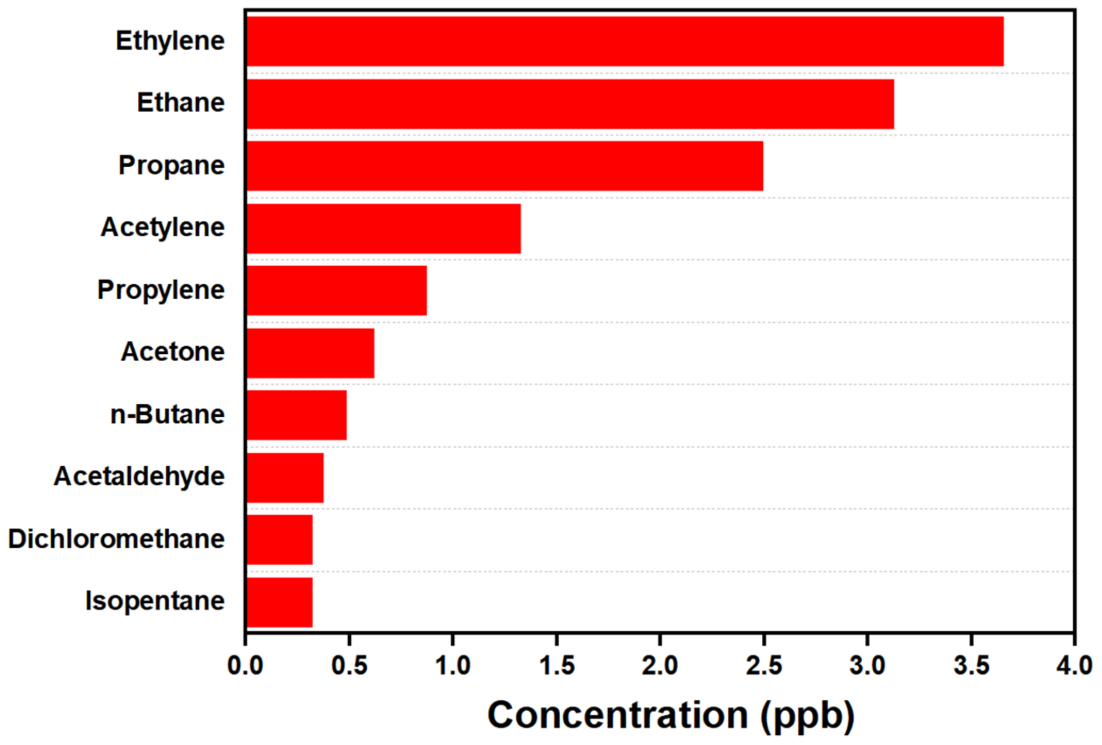

Figure. S2. The top ten VOC species by concentration.

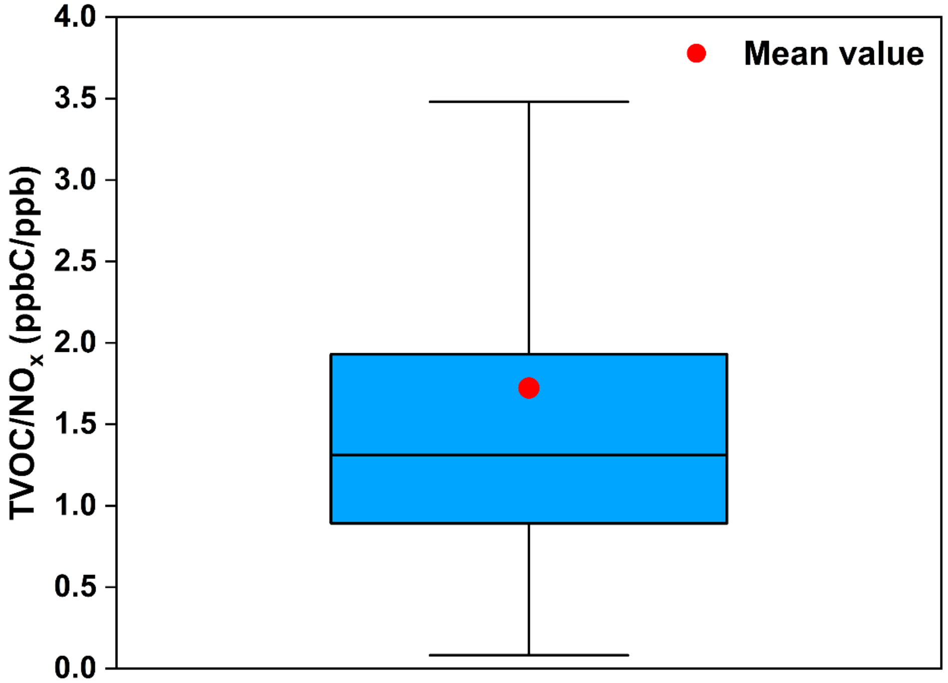

Figure. S3. The daily average ratio of VOCs/NO_x.
